# Borderline pulmonary hypertension is associated with exercise intolerance and increased risk for acute exacerbation in patients with interstitial lung disease

**DOI:** 10.1186/s12890-019-0932-5

**Published:** 2019-09-02

**Authors:** Kenji Nemoto, Shuji Oh-ishi, Tatsuya Akiyama, Yuki Yabuuchi, Hitomi Goto, Mizu Nonaka, Yuika Sasatani, Hiroaki Tachi, Naoki Arai, Hiroaki Ishikawa, Kentaro Hyodo, Isano Hase, Yukiko Miura, Takio Takaku, Kenji Hayashihara, Takefumi Saito

**Affiliations:** Department of Respiratory Medicine, National Hospital Organization, Ibarakihigashi National Hospital, 825, Terunuma. Tokai-mura, Naka-gun, Ibaraki, 319-1113 Japan

**Keywords:** Acute exacerbation, Borderline pulmonary hypertension, Interstitial lung disease, Pulmonary hypertension, 6-min walk test

## Abstract

**Background:**

Pulmonary hypertension (PH) is traditionally defined as a resting mean pulmonary artery pressure (mPAP) of ≥25 mmHg, while mPAP in the range of 21 to 24 mmHg is recognized as “borderline PH.” Interstitial lung disease (ILD) is complicated by the development of PH, which is known to be linked with exercise intolerance and a poor prognosis. Even though it has recently been recommended that PH is redefined as a mPAP of > 20 mmHg, little is known about the clinical significance of borderline PH in ILD. We evaluated whether borderline PH has an impact on the exercise capacity, risk of acute exacerbation (AE), and mortality in patients with ILD.

**Methods:**

A total of 80 patients with ILD who underwent right heart catheterization (RHC) between November 2013 and October 2016 were included. The patients were divided into 3 groups according to the mPAP values: mPAP ≤20 mmHg (No-PH group; *n* = 56), 20 < mPAP < 25 mmHg (Bo-PH group; *n* = 18), and mPAP ≥25 mmHg (PH group; *n* = 6). The demographic, hemodynamic, spirometric, and 6-min walk test (6MWT) data of the patients were collected. In addition, the 1-year incidence of AEs and 1-year survival of the patients after the initial RHC were also evaluated.

**Results:**

There were no significant differences among the 3 groups in the mean age, pulmonary function parameters or the PaO_2_, however, 6-min walk distance was significantly lower in both the Bo-PH and PH groups (*p* < 0.001 for both) as compared to the No-PH group. The results of the Kaplan-Meier analysis revealed that while there was no significant difference in the 1-year survival rate among the three groups, the 1-year incidence of AEs was significantly higher in both the Bo-PH and PH groups (*p* < 0.001, *p* = 0.023, respectively) as compared to the No-PH group.

**Conclusions:**

The current study suggested that borderline PH may be associated with poorer exercise tolerance and an increased risk of AEs in patients with ILD. Therefore, the physicians should pay close attention to the presence of even mild elevation of the mPAP at the initial evaluation in patients with ILD.

## Background

According to the definition based on the 5th World Symposium on Pulmonary Hypertension (WSPH), patients with a resting mean pulmonary artery pressure (mPAP), as assessed by right heart catheterization (RHC), of ≥25 mmHg are defined as having “pulmonary hypertension (PH),” and those with a mPAP of ≤20 mmHg are defined as having “normal pulmonary hemodynamics” [1]. On the other hand, even though a precise classification and management of such patients remained unclear, patients with a mPAP in the range of 21 to 24 mmHg were defined as having “borderline PH” [1]. However, recently, the 6th WSPH was held in Nice, and this task force recommended that the following definition of pre-capillary PH: concurrent presence of a mPAP of > 20 mmHg, pulmonary arterial wedge pressure (PAWP) of ≤15 mmHg, and pulmonary vascular resistance (PVR) of ≤3 WU [2]. Therefore, it is speculated that the clinical evaluation of patients with even mildly elevated mPAP would be more important.

PH is often observed as a complication in patients with interstitial lung disease (ILD), including idiopathic pulmonary fibrosis (IPF), combined pulmonary fibrosis and emphysema (CPFE), and connective tissue disease associated with ILD (CTD-ILD) [3–9]. In patients with IPF, PH is an important complication, since the presence of PH has been shown to be associated with increased dyspnea, deterioration of gas exchange, rapid desaturation on exercise, limitation of exercise capacity as measured by the 6-min walk test (6MWT), increased risk of acute exacerbation (AE), and reduced survival [3–5, 10, 11]. PH is also known to be associated with an increased risk of death in patients with such ILDs as CPFE and CTD-ILD [6–9].

These previous studies related to PH in ILD serve as the basis for defining the cutoff point of the mPAP, as assessed by RHC for defining PH (mPAP ≥25 mmHg). Although some studies have reported poor outcomes in IPF patients with PH defined using other cutoff values (mPAP > 17 mmHg, > 20 mmHg) [12, 13], little is known about the clinical significance of borderline PH in ILD. Therefore, the aim of this study was to evaluate whether borderline PH has an impact on the exercise capacity, risk of AEs, and mortality in patients with ILD.

## Methods

### Subjects

We conducted a retrospective review of consecutive patients with ILD who underwent RHC between November 2013 and October 2016. RHC was performed in subjects with suspected PH based on clinical criteria. The diagnosis of ILD was made based on the clinical findings, serologic findings, and findings of high-resolution computed tomography (HRCT). The diagnosis of IPF was established using standard criteria [14]. Although a surgical lung biopsy was not required for confirmation, in some patients, the diagnosis was established by lung biopsy. We included all patients with CTD-ILD who were diagnosed on the basis of each criteria, however, in whom CTD-associated pulmonary arterial hypertension was excluded based on clinical judgment/multidisciplinary discussion. The exclusion criteria were as follows: (1) patients in whom the initial evaluation was performed using supplemental oxygen; (2) patients suffering from left heart disease, pulmonary arterial thromboembolism, chronic liver disease and obstructive sleep apnea syndrome, all of which could cause secondary PH other than CTD-ILD; (3) PAWP > 15 mmHg on RHC; (4) patients with orthopedic impairment(s) that could interfere with the performance in the 6MWT. According to a previous definition based on the 5th WSPH [1], ILD patients were divided into 3 groups according to the mPAP as measured by RHC: mPAP ≤20 mmHg (No-PH group), 20 < mPAP < 25 mmHg (Bo-PH group), and mPAP ≥25 mmHg (PH group). This study was reviewed and approved by the Ibarakihigashi National Hospital Institutional Review Board (IRB number 2017–022).

### Measurements

We recorded the patients’ characteristics, pulmonary function parameters, KL-6, PaO_2_, the results of the 6MWT, echocardiography and hemodynamics retrospectively. All the patients underwent spirometry (CHESTAC-8900; Chest, Tokyo, Japan) based on the American Thoracic Society (ATS) recommendations for acceptability and reproducibility [15]. Single-breath DLco was also measured (CHESTAC-8900). The values of VC, FEV_1_, TLC and DLco were expressed as percentages of the predicted normal values. The 6MWT was performed in all patients, in accordance with the ATS statement [16]. SpO_2_ was monitored continuously with a wireless pulse oximeter during the 6MWT. If the SpO_2_ fell below 85%, the test was stopped for safety concerns. The distance that the patients could walk in the test was recorded as the 6-min walk distance (6MWD), and the ΔSpO_2_ (initial SpO_2_ – lowest SpO_2_ on 6MWT) was calculated. The right ventricular systolic pressure (RVSP) obtained by echocardiography was measured in all the patients. RHC was performed at rest using a Swan-Ganz catheter inserted percutaneously via either the jugular vein or the femoral vein. Cardiac output was measured by a thermodilution method.

We also evaluated the 1-year incidence of AEs and the 1-year survival after the initial RHC. AE of IPF was defined as previously described [17]. In brief, patients had a previous diagnosis of IPF, and presented with acute worsening of dyspnea within a 1-month period and newly developed bilateral ground-glass opacities and/or consolidation on HRCT, with no evidence of cardiac failure or fluid overload to explain the respiratory deterioration [17]. On the other hand, there is no existing official definition of AE for patients with ILDs other than IPF. However, AEs in patients with ILDs other than IPF resembles the AEs noted in patients with IPF [18]; therefore, we applied the definition of AE in patients with IPF to AE in patients with any kind of ILD.

### Statistical analysis

Data are expressed as means ± standard deviation (SD) or median (interquartile range), as appropriate. Distribution of continuous measurements was evaluated using the Shapiro-Wilk test. To assess the differences among the 3 groups of patients classified as above, we performed one-way analysis of variance (ANOVA), the Kruskal-Wallis test, and the *χ*^2^ test for parametric continuous, nonparametric continuous, and categorical measurements, respectively. The differences among the groups in the 6MWD and ΔSpO_2_ were evaluated using one-way ANOVA, followed by the Tukey-Kramer post hoc test. Using the Kaplan-Meier method and the log-rank test, the impacts of the mPAP on the 1-year incidence of AEs and the 1-year survival were estimated. *p* values of < 0.05 were considered as being indicative of statistical significance. Analysis of all data was performed using SPSS version 24 for Windows (SPSS Inc., Chicago).

## Results

### Patient characteristics

A total of 80 ILD patients were included in our analysis, 23 of whom had IPF (Table 1). Subjects with other ILDs included patients with CTD-ILD (*n* = 15), CPFE (*n* = 8), idiopathic non-specific interstitial pneumonia (*n* = 8), chronic hypersensitivity pneumonia (n = 8), pleuroparenchymal fibroelastosis (*n* = 7), pneumoconiosis (*n* = 2), microscopic polyangiitis-ILD (n = 1), and unclassifiable ILD (n = 8). The numbers of CTD-ILD patients with polymyositis/dermatomyositis (PM/DM), systemic sclerosis (SSc), rheumatoid arthritis (RA) and Sjögren’s syndrome (SjS) were 5 (33.3%), 4 (26.7%), 3 (20.0%) and 3 (20.0%), respectively.

**Table 1:** Clinical characteristics of study population at baseline

Variables	Total	No-PH	Bo-PH	PH	*p-*value*
		mPAP ≤20	20 < mPAP < 25	mPAP ≥25	
Subjects, *N* (%)	80	56	18	6	–
Mean age (years)	71.2 ± 10.3	70.7 ± 11.0	76.7 ± 6.46	73.4 ± 5.32	0.916
BMI (kg/m^2^)	22.5 ± 3.99	21.9 ± 3.38	21.4 ± 2.99	22.7 ± 4.11	0.182
Smoking status, current/former/never	6/40/34	5/25/26	1/10/7	0/5/1	0.352
Underlying disease					
IPF, *N* (%)	23 (28.8)	17 (30.3)	2 (11.1)	4 (66.7)	0.030
CPFE, *N* (%)	8 (10.0)	5 (8.9)	2(11.1)	1 (16.7)	0.822
CTD-ILD, *N* (%)	15 (18.8)	11 (19.6)	4 (22.2)	0 (0)	0.459
Other ILD, *N* (%)	34 (42.5)	23 (41.1)	10 (55.6)	1 (16.7)	0.230
KL-6 (U/ml)	1030(628, 1569)	968 (660, 1448)	965 (420, 1575)	1280(909, 1526)	0.521
PaO_2_(mmHg)	75.4 ± 9.2	76.7 ± 9.3	73.0 ± 9.0	73.1 ± 9.9	0.117
VC, % predicted (%)	74.6 ± 22.9	72.7 ± 22.5	67.9 ± 23.8	79.3 ± 19.0	0.436
FEV_1_, % predicted (%)	90.3 ± 25.3	87.8 ± 26.1	84.5 ± 18.1	93.5 ± 24.5	0.637
TLC, % predicted (%)	76.9 ± 17.7	76.0 ± 16.2	74.3 ± 21.2	78.6 ± 14.4	0.966
DLco, % predicted (%)	50.5 ± 22.6	52.5 ± 24.2	48.2 ± 24.3	41.3 ± 22.6	0.530
6MWD (m)	342.0 ± 130.1	383.8 ± 101.6	235.2 ± 95.3	169.2 ± 140.0	< 0.0001
ΔSpO_2_(%)	7.60 ± 4.06	7.31 ± 4.00	10.0 ± 4.14	9.80 ± 2.28	0.024
RVSP (mmHg)	30 (24, 35)	28 (22.5, 33)	33 (30, 38)	48.5 (38.8, 52.3)	< 0.0001
mPAP(mmHg)	16.5 (14, 21)	15 (13, 17)	21 (21, 22)	27.5 (25.5, 28.8)	< 0.0001
PVR (dynes・sec・cm^−5^)	195 (152, 287)	180 (145, 210)	281 (211, 317)	378 (320, 494)	< 0.0001
Cardiac index(l/min/m^2^)	2.54 (2.27, 2.76)	2.51 (2.21, 2.71)	2.71 (2.54, 3.01)	2.64 (2.17, 2.74)	0.035
PAWP (mmHg)	6.23 ± 2.68	5.93 ± 2.62	7.46 ± 2.96	4.60 ± 1.82	0.056

Data are presented as mean ± standard deviation or median (interquartile range). *p-value is comparison among the three groups (No-PH, Bo-PH, and PH groups). Definition of abbreviations: PH, pulmonary hypertension; BMI, body mass index; IPF, idiopathic pulmonary fibrosis; CPFE, combined pulmonary fibrosis and emphysema; ILD, interstitial lung disease; CTD-ILD, connective tissue disease associated with ILD; KL-6, krebsvon den lungen-6; PaO2, partial pressure of oxygen; VC, vital capacity; FEV1, forced expiratory volume in 1 s;TLC, total lung capacity; DLco, diffusing capacity for carbon monoxide; 6MWD, 6-min walk distance; SpO2, oxygen saturation as measured by pulse oximeter;ΔSpO2, initial SpO2–lowest SpO2on 6-min walk test; RVSP, right ventricular systolic pressure; mPAP, mean pulmonary artery pressure; PVR, pulmonary vascular resistance; PAWP, pulmonary arterial wedge pressure

The mPAP values as assessed by RHC in the 80 patients with ILD were as follows: mPAP ≤20 mmHg (No-PH group; 56 patients (70%)); 20 < mPAP < 25 mmHg (Bo-PH group; 18 patients (22.5%)), and mPAP ≥25 mmHg (PH group; 6 patients (7.5%)). None of the patients were receiving corticosteroids, immunosuppressive agents or vasodilator therapy at the time of the RHC. The clinical characteristics of each groups classified according to the mPAP are summarized in Table 1. There were no differences among the 3 groups in the age, BMI, smoking status, distribution of underlying diseases other than IPF, KL-6, PaO_2_, or measures of pulmonary function. On the other hand, the 6MWD, ΔSpO_2_, RVSP, mPAP, PVR and cardiac index were significantly different among the 3 groups. During the follow-up period after initial evaluation on room air, only 2 patients (Bo-PH group, *n* = 1; PH group, n = 1) received vasodilator therapy (tadalafil alone); 18 patients (No-PH group, *n* = 14; Bo-PH group, *n* = 2; PH group, n = 2) received anti-fibrotic drugs for IPF and 19 patients (No-PH group, *n* = 10; Bo-PH group, *n* = 8; PH group, n = 1) received corticosteroid and/or immunosuppressive therapy; 5 patients (belonging only to the PH group) received long-term oxygen therapy.

### Association between mPAP and 6MWD

**Fig. 1:**
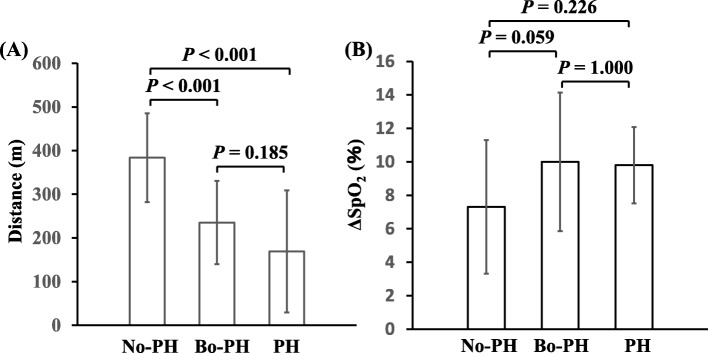
Mean walking distance (m) in the 6MWT (**a**) and ΔSpO_2_ (%) defined by initial SpO_2_ – lowest SpO_2_ on 6MWT (**b**) in the 3 groups of ILD patients. Definition of abbreviations: 6MWT, 6-min walking test; SpO_2_, oxygen saturation as measured by pulse oximeter; PH, pulmonary hypertension

Comparisons of the 6MWD and ΔSpO_2_ among the three groups are shown in Fig. 1. The 6MWD was significantly lower in both the Bo-PH and PH groups than in the No-PH group (*p* < 0.001 for both), however, there was no difference between the Bo-PH group and PH group. Although there were no significant differences in the ΔSpO_2_ among the groups, the ΔSpO_2_ was higher in the Bo-PH group as compared to the No-PH group (*p* = 0.059).

### Association between the mPAP and the 1-year incidence of AE and 1-year survival

**Fig. 2:**
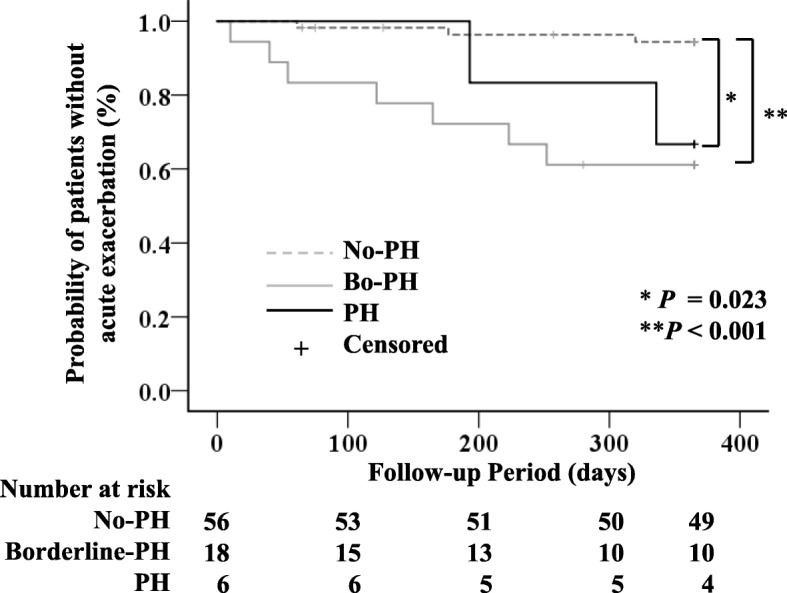
Kaplan-Meier analysis for the onset of acute exacerbation of ILD according to the 3-groups of ILD patients. *Significant difference between No-PH and PH: *p* = 0.023. **Significant difference between No-PH and Bo-PH: *p* < 0.001. Definition of abbreviations: ILD, interstitial lung disease; PH, pulmonary hypertension

**Fig. 3:**
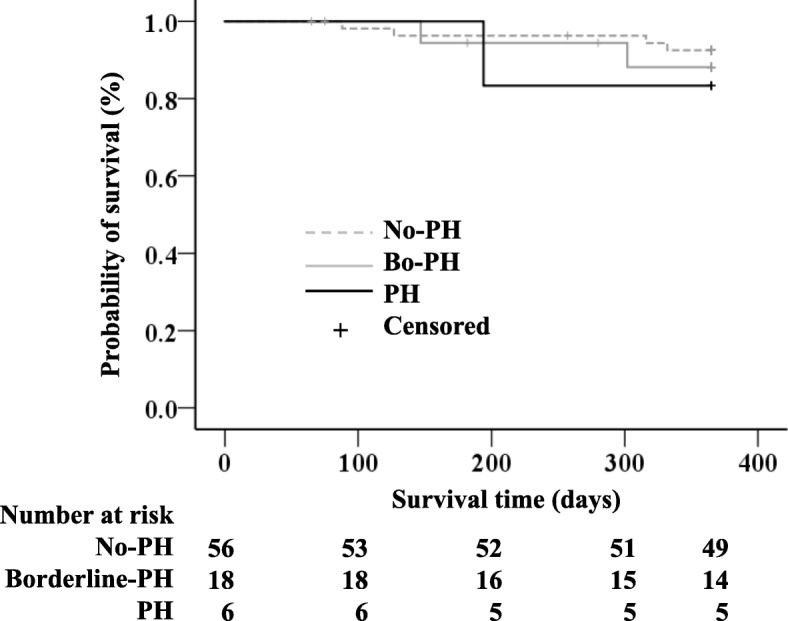
Kaplan-Meier analysis for the 1-year survival according to the 3-groups of ILD patients. There were no significant differences among the 3 groups. Definition of abbreviations: ILD, interstitial lung disease; PH, pulmonary hypertension

Kaplan-Meier analysis showed that the 1-year incidence of AE was significantly higher in both the Bo-PH and PH groups as compared to the No-PH group (p < 0.001, *p* = 0.023, respectively) (Fig. 2). In contrast, the Kaplan-Meier analysis revealed no significant differences in the 1-year survival among the 3 groups (Fig. 3).

## Discussion

The current study shows that not only PH, but also borderline PH, is associated with a decreased exercise capacity and increased risk of AEs in patients with ILD. Therefore, in ILD patients, presence of borderline PH, as defined by a mPAP in the range of 21 to 24 mmHg, should be recognized as a clinical importance similar to PH.

PH is a common complication in patients with ILD [2–8]. Lettieri et al. reported that the prevalence of PH was high (31.6%) in patients with advanced IPF who were referred for lung transplantation [3]. On the other hand, in two different reports of initial evaluation studies which included IPF patients with milder pulmonary function impairment, PH were reported in 8.1 and 14.9% of patients, respectively [12, 13]. In this study, conducted in ILD patients in whom RHC had been performed at the time of the initial evaluation, we found a similar prevalence of PH (13%, 6 of 80 patients) to that in the aforementioned studies.

In regard to the definition of PH, there are only a limited number of studies that have reported the clinical significance of mPAP in the region of the lower cutoff value in patients with ILD. Hamada et al. demonstrated the influence of elevated mPAP (> 17 mmHg) on the prognosis of IPF [12]. Kimura et al. and Suzuki et al. showed that higher values of the mPAP (> 20 mmHg) at the initial evaluation were associated with an increased risk of death in patients with IPF and lung-dominant connective tissue disease, respectively [13, 19]. In addition, Kimura et al. showed that the prognosis seemed to be almost the same between IPF patients with mPAP values in the range of 21–25 mmHg and those with mPAP > 25 mmHg [13]. These studies suggested that a lower cutoff point may be better for defining PH, however, they provided insufficient data to determine the clinical importance of borderline PH in patients with ILD.

Previous studies have documented that, relative to the measures of pulmonary function and hypoxia, altered pulmonary hemodynamics had a greater impact on the 6MWD in patients with IPF [3, 4]. In fact, several studies have shown that the 6MWD was significantly shorter in IPF patients with PH than in IPF patients without PH [3, 4, 11]. In this study, ILD patients with PH had a shorter 6MWD than those with normal pulmonary hemodynamics, consistent with previous reports [3, 4, 11]. Our results also showed that the 6MWD was shorter in the Bo-PH group than that in the No-PH group. These results suggest that not only the presence of PH, but also that of borderline PH, had a significant implication for exercise intolerance in patients with ILD. However, there was no difference in the 6MWD between the Bo-PH group and PH group in this study. In general, higher mPAP was associated with more significant exercise intolerance in patients with IPF [11]. This result could be related to the small sample size and absence of cases with sever disease in our PH group.

In this study, although both the Bo-PH and PH groups seemed to have higher ΔSpO_2_ values (initial SpO_2_ – lowest SpO_2_ on 6MWT) as compared to the No-PH group, there were no significant differences among the 3 groups. These results are in contrast to the previous report that the lowest SpO2 during the 6MWT was significantly lower in IPF patients as compared to that in patients without PH [3]. This discrepancy may be explained by the difference in the 6MWT, which was performed under the local rule that it was stopped, owing to safety concerns, if the SpO_2_ fell below 85%. In this study, there were 9/56 (16%) in the No-PH group, 8/18 (44%) in the Bo-PH group, and 5/6 (83%) in the PH group in whom this rule was applied. Therefore, the ΔSpO_2_ in both the Bo-PH and PH groups in our study may have been underestimated as compared to that in the No-PH group.

In this study, among the 80 patients who underwent RHC during their initial workup, the 1-year incidence of AE after RHC was 21.3% (17 patients). On the other hand, Song et al. reported that the 1-year incidence of AE was 14.2% in a retrospective review of 461 patients with IPF [20]. However, this study included subjects with milder pulmonary function impairment (mean %FVC > 72%, mean %TLC > 73.8%, mean %DLco > 62.2%) than those in our study, and the presence of PH at the baseline was not evaluated [20]. Therefore, it is possible that the higher incidence of AE in our study might be due to fact that our patients had a relatively greater severity of ILD when they underwent the initial workup.

Our study revealed that the presence of PH at the baseline was associated with an increased risk of AEs in patients with ILD. This result was consistent with those reported by Judge, who showed that PH was an independent predictor of the development of AEs in patients with advanced IPF [10]. There are insufficient data about the mechanism by which PH increases the risk of AEs in patients with ILD. Previous reports have shown that the pathogenetic mechanisms of PH in cases of IPF include hypoxic vasoconstriction, destruction of the pulmonary capillaries, and vascular remodeling mediated by various growth factors, such as vascular endothelial growth factor, platelet-derived growth factor and transforming growth factor-β [4, 5, 21]. Although the etiology of AE remains uncertain, Collard et al. suggested that the pathobiology of AE in patients with IPF involves both acceleration of the underlying chronic factors contributing to the fibrotic process and acute factors that lead to widespread acute lung injury [17]. Various growth factors which are related to the development of PH are also likely intrinsic factors that cause progression of the underlying fibrotic condition; therefore, the presence of PH might have increased the risk of AE in this study. We also showed that the 1-year incidence of AEs was significantly higher in the Bo-PH group than in the No-PH group. Therefore, these results seem to confirm that the presence of a mPAP of > 20 mmHg was redefined as an initial pulmonary vasculopathy in patients with ILD [2].

In our study, there were no differences in the 1-year survival among the 3 groups. This result was in contrast to previous reports [3, 13]. Lettieri et al. reported that the 1-year mortality rate was significantly greater in IPF patients with PH who were listed for lung transplantation as compared to those without PH [3]. Kimura et al. divided the IPF patients undergoing RHC into 3 groups (mPAP ≤20 mmHg, 21–25 mmHg, and > 25 mmHg) and compared the 5-year survival according to the mPAP [13]. In their study, significant differences in the mortality were demonstrated among the 3 groups, and patients with mPAP in the range of 21–25 mmHg and > 25 mmHg had higher mortality rates [13]. This discrepancy may be explained by differences in the underlying diseases, sample size, and follow-up period. Furthermore, in this study, the treatment decision after the initial work-up was left to the discretion of the treating physician and thus of limited homogeneity. Therefore, further studies will be necessary to elucidate the association between the presence of borderline PH and mortality in ILD patients.

Our study has several limitations. First, this is a retrospective study with small number of patients. The sample size in this study seemed to confer the low statistical power to detect significant survival indicate. While the size of our study population is limited, the fact that ILD patients with borderline PH had a shorter 6MWD and higher risk of AEs than those with normal pulmonary hemodynamics leads to a good rationale for clinical significance of borderline PH. Second, our study included only initial evaluations performed on room air. Since the need for supplemental oxygen is common among patients with advanced ILD, the severe cases of ILD may have been excluded from this study. However, 6MWT may be influenced by supplemental oxygen therapy, because of an improvement of exercise-induced hypoxic pulmonary vasoconstriction. In fact, previous studies of the 6MWT have also excluded individuals using supplemental oxygen therapy [3, 13].

## Conclusions

The current findings suggest that in ILD patients, the presence of borderline PH, as defined by a mPAP in the range of 21 to 24 mmHg, may be associated with poorer exercise tolerance and an increased risk of AEs, similar to the presence of PH. Therefore, it is recommended that physicians should pay attention to even mild elevation of the mPAP at the initial evaluation in patients with ILD.

## Data Availability

The datasets used and/or analysed during the current study are available from the corresponding author on reasonable request.

## Abbreviations

6MWD: 6-min walk distance
6MWT: 6-min walk test
AE: Acute exacerbation
ATS: American Thoracic Society
CPFE: Combined pulmonary fibrosis and emphysema
CTD-ILD: Connective tissue disease associated with ILD
DLco: Diffusing capacity for carbon monoxide
FEV_1_: Forced expiratory volume in 1 s
HRCT: High-resolution computed tomography
ILD: Interstitial lung disease
IPF: Idiopathic pulmonary fibrosis
KL-6: Krebs von den lungen-6
mPAP: Mean pulmonary artery pressure
PaO_2_: Partial pressure of oxygen
PAWP: Pulmonary arterial wedge pressure
PH: Pulmonary hypertension
PM/DM: Polymyositis/dermatomyositis
PVR: Pulmonary vascular resistance
RA: Rheumatoid arthritis
RHC: Right heart catheterization
RVSP: Right ventricular systolic pressure
SD: Standard deviation
SjS: Sjögren’s syndrome
SpO_2_: Oxygen saturation as measured by pulse oximeter
SSc: Systemic sclerosis
TLC: Total lung capacity
VC: Vital capacity
WSPH: World Symposium on Pulmonary Hypertension
